# Metabolic Footprint Analysis Uncovers Strain Specific Overflow Metabolism and D-Isoleucine Production of *Staphylococcus Aureus* COL and HG001

**DOI:** 10.1371/journal.pone.0081500

**Published:** 2013-12-03

**Authors:** Kirsten Dörries, Michael Lalk

**Affiliations:** Institute of Biochemistry, Ernst-Moritz-Arndt-University of Greifswald, Greifswald, Germany; University Hospital Münster, Germany

## Abstract

During infection processes, *Staphylococcus aureus* is able to survive within the host and to invade tissues and cells. For studying the interaction between the pathogenic bacterium and the host cell, the bacterial growth behaviour and its metabolic adaptation to the host cell environment provides first basic information. In the present study, we therefore cultivated *S. aureus* COL and HG001 in the eukaryotic cell culture medium RPMI 1640 and analyzed the extracellular metabolic uptake and secretion patterns of both commonly used laboratory strains. Extracellular accumulation of D-isoleucine was detected starting during exponential growth of COL and HG001 in RPMI medium. This non-canonical D-amino acid is known to play a regulatory role in adaptation processes. Moreover, individual uptake of glucose, accumulation of acetate, further overflow metabolites, and intermediates of the branched-chain amino acid metabolism constitute unique metabolic footprints. Altogether these time-resolved footprint analyses give first metabolic insights into staphylococcal growth behaviour in a culture medium used for infection related studies.

## Introduction

*Staphylococcus aureus* as an important human pathogen causes not only severe acute nosocomial and community-acquired infections; it also shows increasing resistance to common antibiotics [1], [2]. Within the last years, *S. aureus* has become a subject of intensive research. Because of its ability to invade host cells [3], [4], [5], infection biology, with a focus on the interaction of the eukaryotic host cell and the pathogenic bacterium, is of major interest [6], [7], [8]. With regard to the infection studies, the RPMI 1640 medium is commonly used for the cultivation of eukaryotic cell lines as e.g. epithelial cell lines and macrophages, which are natural counterparts of *S. aureus* during infection processes. Necessary for a bacterial infection study is the precultivation of the bacterium, mostly as a shake-flask culture. Following this, a given number of bacterial cells are added to the eukaryotic cell system, initiating the infection event. For the precultivation of the bacteria, the usage of complex media like LB [6], [9], [10], as well as eukaryotic cell culture media is common [11], [12]. The latter offers three benefits i) the bacteria are not forced to adapt to drastically changed nutritional supply, ii) time consuming washing steps are needless and iii) the bacterial stress response is being kept to a minimum within the experimental setup. Its importance becomes more obvious when taking into account, that changes in the environment, amongst others, have impact on the metabolic status which in turn influences virulence and pathogenicity of the bacterium [13], [14], [15]. Consequently, each culture medium used in laboratory research causes individual adaptive processes and growth behaviour of the bacterium [16], leading to divergent results depending on each varying nutritional supply [17]. Considering the strong influence of the culture medium on the bacterial physiology, we investigated the exometabolome of *S. aureus* during growth in the RPMI 1640 medium. Therefore we used the *S. aureus* strains COL and HG001. Both strains are sequenced and commonly used strains in laboratory for basic research. HG001 is a *rsbU* repaired derivate of the strain NCTC 8325, which is a MSSA isolate [18] and COL is a clinical MRSA isolate [19]. Differences in e.g. staphyloxanthin formation and exoprotein expression have already been observed for COL and HG001 [18], [20]. This gives reason for the assumption that exometabolome data may highlight even more diversity between these two strains.

By using ^1^H-NMR spectroscopy, we analyzed the metabolic footprints [21] along the growth curve of *S. aureus* COL and HG001. This provides first insights into the growth behaviour and basic physiological processes including remarkable secretion profiles of COL and HG001 cultivated in a basic eukaryotic cell culture medium. A separation of COL and HG001, based on their time-resolved exometabolic profiles, could be obtained, giving important information for future infection studies.

## Materials and Methods

### Bacterial strains and growth conditions

*S. aureus* COL [19] and HG001 [18] were grown in RPMI 1640 R7509 medium (Sigma-Aldrich, St. Louis, USA) for both overnight and main culture. 2 mmol/l glutamine was added as prescribed by the manufacturer. FeCl_3_ and trace elements were also added [22]. Bacterial cultures were grown aerobically with vigorous agitation at 130 rpm at 37°C, and with a liquid-to-air ratio of 1∶5. Aerobic growth was previously verified for these culture conditions by Fuchs and coworkers [23].

### Sampling for extracellular metabolome analysis

The main culture was inoculated with an exponentially growing overnight culture to an initial optical density at 500 nm of 0.06. Every 90 minutes, the optical density was monitored and 2 ml cell suspension were filtered on ice by using a 0.45 µm pore size filter (Sarstedt AG, Nürnberg, Germany), to get sterile extracellular metabolite samples of the bacterial culture. All filtrates were stored at −20°C before measurement. The experiment was carried out in 4 independent biological replicates.

### ^1^H nuclear magnetic resonance (^1^H-NMR) spectroscopic analysis of extracellular metabolites

^1^H-NMR analysis was done in 5 mm glass tubes (7 inch in length; NORELL ST-500, NORELL, Inc., USA). 400 µl sample volume were buffered to a pH of 7.0 by adding 200 µl of a 0.2 mol/l sodium hydrogen phosphate buffer solution, made up with 50% D_2_O (Euriso-Top, St-Aubin Cedex, France), which provides a NMR-lock signal. Additionally, the buffer solution contained 1 mmol/l TSP (3-trimethylsilyl-[2,2,3,3-D4]-1-propionic acid) (Sigma-Aldrich, St. Louis, USA) as an internal standard for subsequent quantification. All NMR spectra were obtained at 600.27 MHz at a temperature of 310 K using a Bruker AVANCE-II 600 NMR spectrometer, operated by TOPSPIN 2.1 software (both from Bruker Biospin GmbH, Rheinstetten, Germany). A 1D-NOESY pulse sequence was used with presaturation on the residual HDO signal during both the relaxation delay and the mixing time. A total of 64 free induction decays (FID scans) were collected, using a spectral width of 30 ppm for a one-dimensional spectrum. Spectral referencing was done relative to the TSP signal.

### ^1^H-NMR data analysis

Data analysis (identification and quantification) was done by using AMIX-Viewer v3.9.11 software (Bruker Biospin GmbH, Rheinstetten, Germany). The identification based on spectra alignment of pure standard compounds (Sigma-Aldrich, St. Louis, USA). 2-acetolactate was synthesized by saponification of 1 mol ethyl 2-acetoxy-2-methylacetoacetate (Sigma-Aldrich, St. Louis, USA) and 2 mol NaOH. Quantification was done by integration and comparison of designated peaks to the TSP signal, considering the amount of protons of each signal. Unidentified signals were relatively quantified (integrated metabolite signal referred to integrated TSP signal) due to their unknown quantity of protons. Each unknown metabolite was named according to its chemical shift (ppm) and its signal multiplicity in the NMR spectra.

### pH measurement

During cultivation of COL and HG001, the pH value was determined at each sampling time point of 4 biological replicates by using HI 2211 pH/mV/°C bench meter (HANNA instruments Deutschland GmbH, Kehl, Germany).

### Visualization and statistical analyses

Experimental data were displayed as the mean of quadruplicate samples with its standard deviation. Visualization of time dependent changes in concentrations of extracellular metabolites was done by using VANTED v2.01 [24] and Microsoft Excel 2007. Statistical significant differences were calculated by using unpaired T-test with *p≤0.01 using VANTED v2.01 [24]. Statistical separation via principal component analysis (PCA), including the loading plot and their visualization, were done by using PAST v2.15 [25] with the default settings. An overview of all identified extracellular metabolites via a hierarchical clustered heatmap was created by using MeV v4.8.1 [26] with the following settings: optimized gene leaf order, euclidean distance metric and average linkage method. For the tree configuration, a 0.022 distance threshold was adjusted with 50 terminal nodes.

## Results and Discussion

### Phenotypes and statistical separation

**Figure 1:**
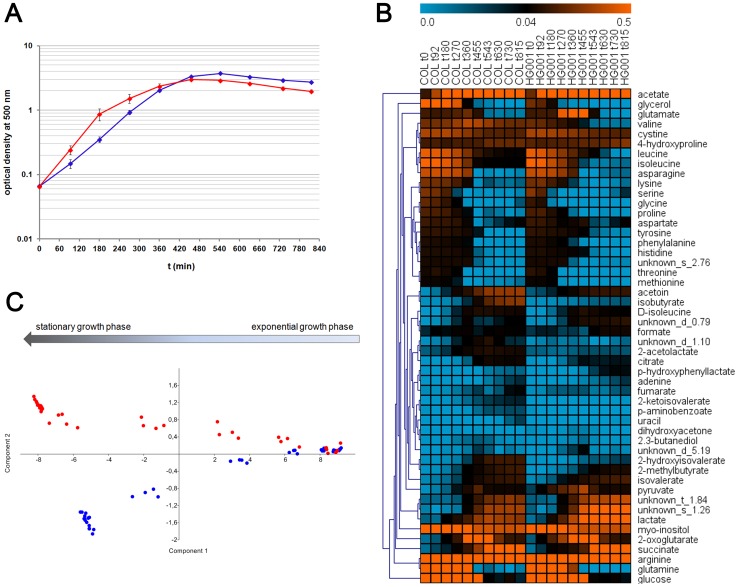
Growth curves, extracellular metabolites and the statistical separation of *S. aureus* COL and HG001. (**A**) Growth curves of COL (blue line) and HG001 (red line) in RPMI medium are presented. Data are shown as mean values ± SD of quadruplicate samples. (**B**) Time-resolved extracellular metabolite concentrations (mmol/l) of COL and HG001 were visualized with MeV as color coded chart. Absent or low concentrated metabolites are displayed in blue, whereas increasing concentration turns into orange coloration. Concentrations greater than 0.5 mmol/l are colored in orange. Changes of yet unknown metabolites are displayed based on relative concentrations. Metabolites are arranged by hierarchical cluster analysis. Displayed are the mean values of concentrations of 4 biological replicates. (**C**) Principal component analysis of the exometabolome data of *S. aureus* COL (blue points) and HG001 (red points) of ten sampling time points. Single values of 4 biological replicates are displayed. The arrow indicates the starting and end points of the time-resolved exometabolome data. The percentages of variance are 97.407 for component 1 and 1.899 for component 2.

**Figure 2:**
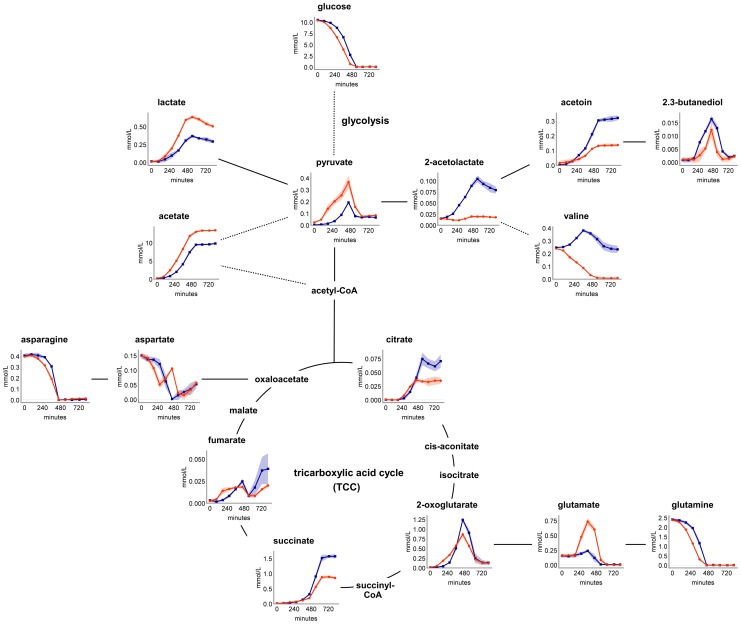
Overflow metabolites and associated metabolic pathways. Time-resolved extracellular metabolite concentrations of COL (blue) and HG001 (orange) are arranged according to their intracellular metabolic pathways as they are glycolysis, pyruvate metabolism, TCC and glutamine and asparagine metabolism. Dashed lines represent multiple successional enzymatic reactions. Data are shown as mean values ± SD of quadruplicate samples. SD is shown as color shading.

**Figure 3:**
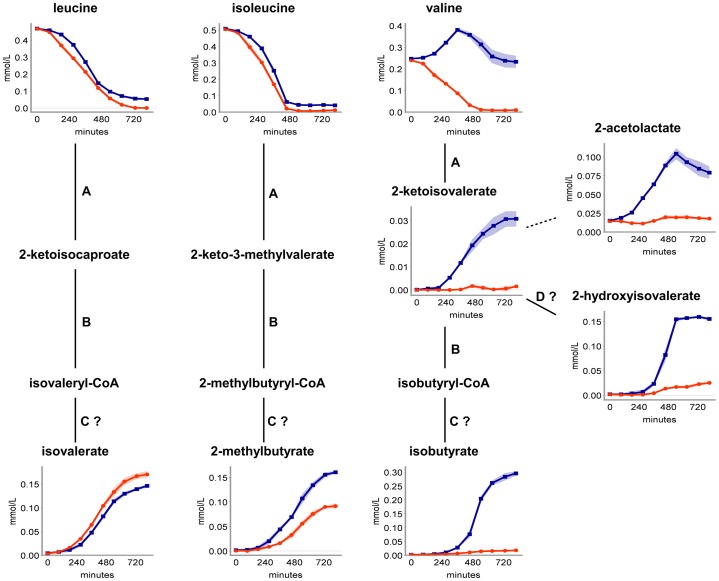
BCAA metabolism. Extracellular concentration profiles of branched-chain amino acids and their detected degradation products of *S. aureus* COL (blue) and HG001 (orange) are presented. Involved enzymes are abbreviated as follows: A (branched-chain amino acid aminotransferase), B (dehydrogenase complex), C (acyl-CoA hydrolase) and D (reductase), whereby C and D are signed with question marks indicating possible enzymatic reactions. Dashed lines represent multiple successional enzymatic reactions. Data are shown as mean values ± SD of quadruplicate samples. SD is shown as color shading.

Using RPMI as a cultivation medium, a well reproducible growth of *S. aureus* was obtained (Figure 1A), which is reflected by likewise reproducible exometabolic profiles (Figures 2, 3). By using ^1^H-NMR analysis, we quantified all metabolic components of the RPMI medium, except for tryptophan and the vitamins, which concentrations were below the detection limit of the analytical method. Furthermore, we detected secreted metabolites in the supernatant of *S. aureus* COL and HG001; altogether resulting in 44 identified and absolutely quantified metabolites and 6 unidentifiable and relatively quantified metabolites (Figure 1B, Table S1, Table S2). These yet unknown metabolites were named unknown s_1.26, s_2.76, d_0.79, d_1.10, d_5.19 and t_1.84. Still, identification of these metabolites should be accomplished in further studies, since they show interesting uptake and secretion patterns. By using the extracellular metabolic data of COL and HG001, both strains could be separated statistically via principal component analysis (Figure 1C). Thereby, the changes in the glucose concentration and the acetate concentration had the highest impact on the separation (Figure S1A). The different uptake and secretion rate of both metabolites are also visible when plotting their concentration as a function of growth (Figures S1B, S1C). This plotting of concentrations against equivalent optical densities demonstrates, that HG001 takes up glucose in a faster way than COL does, and additionally HG001 secretes acetate faster and in higher amounts than COL does. Regarding both phenotypes during aerobic cultivation in RPMI, COL has a higher growth yield compared to HG001, but in contrast HG001 has a higher growth rate during exponential growth (Figure 1A). The different growth rates of both strains have also been observed when cultivating *S. aureus* COL and HG001 in a complex medium [18]. The reason for the lower growth rate of COL is still unknown, but in this study it is associated with a slower glucose uptake. Besides glucose and acetate, the secretion profiles are also determined by other metabolites, resulting in strain specific metabolic footprints (Figures 1B, 1C). This already points to the different routes within the central carbon metabolism of both strains which are presented in the following.

### Glucose consumption and overflow metabolites

Under aerobic conditions and excess of glucose, the latter is catabolized incompletely, mainly via glycolysis [27] to pyruvate, which is subsequently forced into the overflow metabolism due to the surplus of carbon and energy. Under these conditions, acetate is the most prominent overflow metabolite [28], [29]. This is in accordance with our results, since COL accumulated 9.8 mmol/l acetate and HG001 accumulated 13.4 mmol/l acetate in the medium (Figure 2). In this study, further overflow metabolites were detected for both strains, as they are lactate, pyruvate, 2-acetolactate, acetoin, and 2.3-butanediol (Figure 2). Compared to acetate, the other overflow metabolites are secreted in notably smaller quantities, all below 1 mmol/l. Interestingly, COL secreted 2-acetolactate, acetoin, and 2.3-butanediol in significantly (*p*≤0.01) higher concentrations compared to HG001. In contrast, HG001 secreted significantly (*p*≤0.01) larger amounts of acetate, lactate, and pyruvate, compared to COL (Figure 2). These observations contributed to the statistical separation of both strains, as mentioned above.

A benefit of the conversion of pyruvate to the branching overflow pathways is the regeneration of NAD^+^ participating in the intracellular balancing of the redox equivalents NADH/NAD^+^, which has been studied extensively for *Lactococcus lactis* [30], [31], [32]. Both strains seem to use the overflow pathways in varying proportions resulting in the different pattern of accumulated overflow metabolites in the culture supernatant.

### Tricarboxylic acid cycle intermediates

During exponential growth with available glucose in the medium, the tricarboxylic acid cycle (TCC) is predicted to be weakly active [33]. This is in accordance with the accumulation of citrate, 2-oxoglutarate, succinate, and fumarate during the exponential growth of COL and HG001 (Figure 2). Succinate and 2-oxoglutarate reached concentrations up to 1 mmol/l in the supernatant, thereby exceeding extracellular concentrations of several common overflow metabolites, like lactate and acetoin. Interestingly, COL secreted significantly (*p*≤0.01) higher amounts of citrate, 2-oxoglutarate, and succinate compared to HG001. These observations may point to minimal activity of single TCC enzymes during exponential growth of COL and HG001. This would be in line with several findings of previous studies, in which TCC proteins were detectable [29], [34] and also the activity of several TCC proteins was verified [33], [35], [36] in exponentially growing cells and with glucose available in the medium. Additionally, an intracellular pool of TCC intermediates could be found under these conditions [29].

### Uptake of overflow and central carbon metabolites

The reuse of pyruvate and 2-oxoglutarate started simultaneously when glucose was depleted (Figure 2) pointing to their role as important central metabolites which are necessary for many different metabolic pathways. Further metabolites that were taken up again are 2.3-butanediol, lactate, fumarate and 2-acetolactate, whereas 2-acetolactate was only secreted and taken up in an appreciable amount by COL (Figure 2). These metabolites may serve as alternative carbon sources and they can fill up central metabolic pathways. An exception is fumarate. It was secreted again simultaneously to aspartate which will be discussed below.

Interestingly, when glucose is depleted, no uptake of acetate, succinate, citrate, and acetoin was detectable for both strains (Figure 2). Since acetate can serve as an alternative carbon source when glucose is depleted, its remaining in the supernatant is a noticeably outcome of this study. Considering that the RPMI medium is pH buffered, the accumulation of acetate had no noticeable effect on the extracellular pH (Figure S2). In turn, the adjusted extracellular pH value of the stationary phase culture was at around 8.5 for both strains. This is in accordance with the results of previous studies using unbuffered media, in which the pH of the stationary phase cultures of *S. aureus* increased up to almost the same value [13], [36], [37]. In these studies, the re-alkalization of the medium was depending on the acetate uptake, since it is the main overflow metabolite under aerobic growth conditions. We can only speculate whether the absent acetate utilization is related, among others, to the stable pH conditions in our study. However, our results are in line with previous findings, where no uptake was observable during an aerobic cultivation of *S. aureus* in a pH buffered LB medium [38].

### Amino acid uptake and secretion

Besides glucose, a large number of amino acids are available in the RPMI medium. They were already taken up by both strains during the exponential growth phase, except 4-hydroxyproline (Figure 1B). After 13 hours of cultivation, both strains nearly used up all the amino acids, except for arginine, cystine, 4-hydroxyproline and aspartate. Additionally, the branched-chain amino acids (BCAAs) were still detectable in the supernatant of COL in the stationary growth phase. In this study, besides the common overflow metabolites, we detected several secreted metabolites which most probably resulted from amino acid degradation reactions and which profiles differ between both *S. aureus* strains.

### Glutamine as nitrogen source

Glutamine and to a lesser extent asparagine are certainly the main nitrogen donors, since no NH_4_^+^ as a nitrogen source is available in the RPMI medium. Remarkably, in the mid-exponential growth phase, HG001 showed a significant (*p*≤0.01) secretion of glutamate, starting from 0.15 up to 0.74 mmol/l, and in parallel the aspartate concentration increased although aspartate was taken up before (Figure 2). In contrast, COL solely secreted glutamate in small amounts up to 0.24 mmol/l. Glutamate is the most abundant amino acid in the cytoplasm of *S. aureus* [29], but nevertheless the bacteria attempt to maintain an equilibrium intracellularly, which is controlled among others by the TCC [16]. Glutamine is the main nitrogen source and the highest concentrated amino acid in the RPMI medium. Its deamidation reaction will rapidly fill up the intracellular glutamate pool. In order to maintain the equilibrium of glutamate, COL and HG001 behaved differently. HG001 tended to secrete excess glutamate and also aspartate, resulting in the accumulation of both amino acids in the supernatant during exponential growth (Figure 2). COL took up both nitrogen sources glutamine and asparagine in the same way that HG001 did, but COL only secreted glutamate sparsely. Keeping in mind that COL secreted TCC intermediates (citrate, 2-oxoglutarate and succinate) in higher quantities compared to HG001, we suggest that glutamate and aspartate were metabolized further, probably by entering into the TCC. Besides the TCC, glutamate and aspartate can also be integrated in further metabolic pathways as the purine and pyrimidine biosynthesis and the urea cycle.

When glucose, glutamine and asparagine were depleted extracellularly, both glutamate and aspartate were taken up again (Figure 2). This may be due to the induced TCC enzymes and amino acid degradation enzymes, by which amino acids can be used as alternative carbon sources when glucose is depleted [39]. This is in accordance with the simultaneous uptake of 2-oxoglutarate and fumarate (Figure 2). Why aspartate has been secreted in the stationary growth phase, remains unclear. We can only speculate whether this is associated with a stop in pyrimidine synthesis for which aspartate serves as a precursor, since we also detected secretion of uracil in the stationary growth phase (Figure 1B, Table S1). Additionally, a clear accumulation of adenine, starting in the transient growth phase of both strains, supports this idea (Figure 1B, Table S1).

### Branched-chain amino acid metabolism

During growth of *S. aureus*, downstream products of BCAA degradation accumulated in the culture supernatant (Figure 3). COL and HG001 produced isovalerate and 2-methylbutyrate, whose amounts represent at least 20% of the initial leucine and isoleucine concentration of the RPMI medium. This accumulation may be due to the fact that not all BCAAs are used up for protein biosynthesis, although BCAAs are the most abundant amino acids in staphylococcal proteins (http://cmr.jcvi.org/cgi-bin/CMR/shared/GenomePropertiesHomePage.cgi). For *S. aureus*, BCAAs, especially isoleucine, are known to be effectors of CodY [14], [15], [40], which is a global regulator necessary for the adaptation to nutrient limitations. Therefore the maintenance of an intracellular equilibrium of the BCAAs seems to be important for the growth of *S. aureus*, which is in line with the findings for *Lactococcus lactis* [41] and *Bacillus subtilis* (H. Meyer, unpublished data). For maintaining this equilibrium, catabolic reactions via the branched-chain amino acid aminotransferase are probably necessary. During this transamination step, branched-chain keto acids and glutamate are generated, thus redundant BCAAs may additionally serve as nitrogen sources. 2-Oxoglutarate is needed as nitrogen acceptor, confirming its role as a central metabolite. Studies on *Saccharomyces cerevisiae* showed up different routes from the branched-chain keto acids to 2-hydroxyisovalerate, isobutyrate, isovalerate and 2-methylbutyrate [42], [43], [44]. Further, in yeasts, the carbon skeletons derived from the BCAAs do not enter the TCC but end up in the so-called “fusel” alcohols [44]. Since no uptake of the BCAA degradation products was detected for both staphylococcal strains, this provides the evidence, that COL and HG001 do not use the carbon skeletons of BCAAs for filling up the TCC, but produce different branched-chain organic acids which accumulate in the medium.

Interestingly, valine and intermediates of its synthesis and degradation, as they are 2-acetolactate, 2-ketoisovalerate, 2-hydroxyisovalerate and isobutyrate, were significantly (*p*≤0.01) more secreted by COL into the medium (Figure 3). This represents a distinguishing characteristic regarding both strains, although COL and HG001 possess the same genetic makeup for BCAA synthesis and degradation, as well as branched-chain fatty acid synthesis. The increase in concentration of 0.14 mmol/l gives a hint to ongoing valine biosynthesis during exponential growth of COL, although the genes for valine biosynthesis are under CodY control [14], [15] and valine was still available in the medium. The hypothesis of valine biosynthesis is supported by the similar secretion and uptake profile of 2-acetolactate (Figure 3), since this metabolite is also an intermediate in valine biosynthesis. A side effect is the incorporation of two molecules pyruvate and the requirement of one molecule glutamate as nitrogen donor for building up one molecule valine, contributing to a lowering of the intracellular pyruvate and glutamate pool. Additionally, one molecule NAD(P)^+^ is being regenerated by the reductoisomerase IlvC. Subsequently, valine was taken up by COL simultaneously to pyruvate, 2-acetolactate, 2.3-butanediol, 2-oxoglutarate and glutamate. The valine derivates remained in the supernatant similar to the degradation products of leucine and isoleucine. Whether 2-acetolactate is a by-product of 2.3-butanediol or valine biosynthesis remains unclear.

### D-isoleucine accumulation

**Figure 4:**
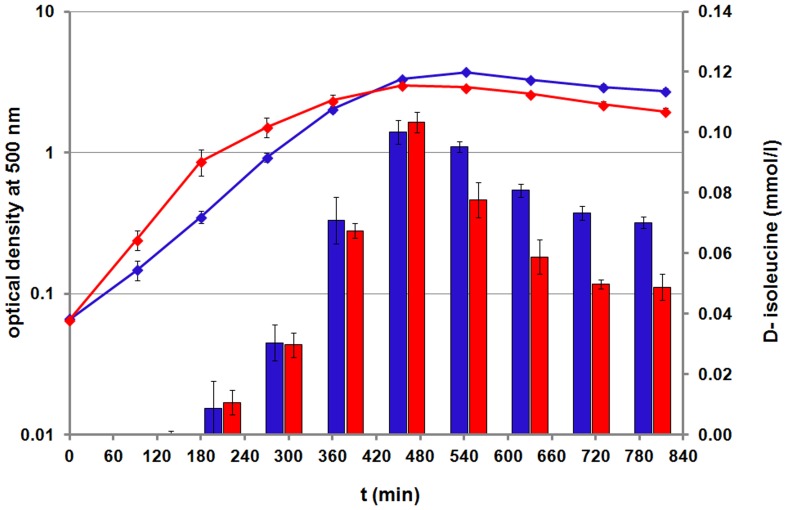
D-isoleucine accumulation. The D-isoleucine concentration in the supernatant of both strains is presented in blue columns for COL and in red columns for HG001. The according growth curves are shown as blue (COL) and red (HG001) lines. Data are shown as mean values ± SD of quadruplicate samples.

An outstanding result is the detection of D-isoleucine in the supernatant of both staphylococcal strains (Figure 4). Recently, many bacteria were found to produce non-canonical D-amino acids (NCDAAs), probably by several putative racemases [45]. NCDAAs were found to play a regulatory role in adaptation to changing environmental conditions and to have an impact on the cell wall composition of bacteria by getting incorporated in peptidoglycan polymers [45], [46]. This incorporation can arise via different mechanisms and in the intracellular or extracellular space [47], [48]. Extracellularly racemized D-amino acids can be transported into the cytoplasm, where they may serve as substrates for the dipeptide formation via ligases, and so step into cell wall synthesis. Besides, in the extracytoplasmic space, D-amino acids can be incorporated into the muropeptides of peptidoglycan subunits during the assembly process by modifying enzymes like penicillin-binding proteins [47]. For *Bacillus subtilis*, it was shown that the incorporation takes place mainly in the extracytoplasmic space [47]. Additionally, studies on a purified *Escherichia coli* transpeptidase showed that many structural different NCDAAs serve as substrates for this enzyme [48]. For *S. aureus*, NCDAAs were found to inhibit biofilm formation in liquid medium [49], [50]. Lam and coworkers (2009) analyzed the supernatant of stationary *S. aureus* cells grown in LB medium, and they detected mostly D-alanine (1.52 mmol/l) and D-leucine, D-isoleucine, D-proline and D-tyrosine in lower quantities (≥0.05 mmol/l) [45]. Moreover, they assumed that the accumulation of NCDAAs is a phenomenon of bacterial cells in the stationary growth phase [45].

For the first time, we can show the time dependent accumulation of extracellular D-isoleucine. This accumulation already started during the exponential growth, reaching a maximum of about 0.1 mmol/l, when bacterial cells entered the stationary growth phase (Figure 4). D-isoleucine accumulation stopped at the same time when L-isoleucine was almost depleted in the supernatant. Afterwards, a slow decrease in concentration, simultaneous to the decrease in optical density, took place. This may be a result of an ongoing incorporation of D-isoleucine into peptidoglycan, starting in the transient growth phase. Whether D-isoleucine is racemized extra- or intracellularly still remains unclear, but it can also be detected in the cytosol (data not shown). Our data also suggest, that growth in a chemical defined medium in contrast to growth in a complex medium, probably first results in a smaller spectrum of NCDAAs, as we could only detect one D-amino acid, and second in lower concentrated NCDAAs. Nevertheless, 20% of the initial L-isoleucine concentration of 0.5 mmol/l is getting conversed into its D-configuration. This accumulation of D-isoleucine may be an interesting starting point for further research on its potential incorporation into the cell wall or any inhibitory function on biofilm formation in shaking cultures.

### 4-hydroxyphenyllactate production

**Figure 5:**
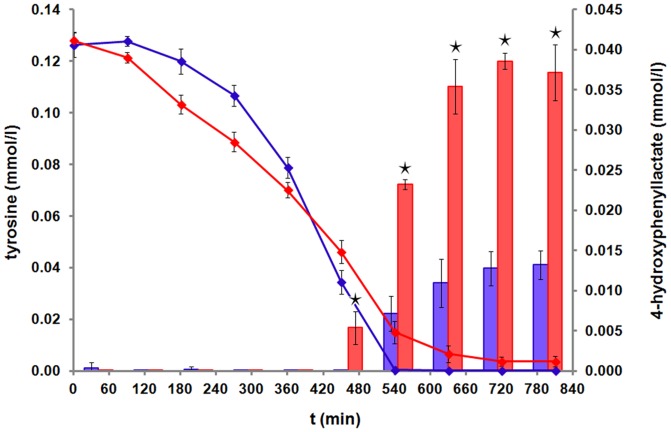
Secretion of 4-hydroxyphenyllactate. The tyrosine uptake during growth in RPMI medium is presented as a blue line for COL and a red line for HG001. 4-OH-PLA secretion is displayed as blue columns for COL and red columns for HG001. Data are shown as mean values ± SD of quadruplicate samples. Statistical differences between COL and HG001 were considered as significant with *p≤0.01.

Several lactic acid bacteria, which are used as natural biopreservatives of many food products, were found to produce the antifungal organic acids phenyllactate and 4-hydroxy-phenyllactate (4-OH-PLA) [51], [52]. In the culture supernatant of both *S. aureus* strains, a production of 4-OH-PLA was observed, started in the transient growth phase (Figure 5). Tyrosine needs to be available in the culture medium for the synthesis of 4-OH-PLA [52] which is in accordance with the tyrosine uptake by both strains (Figure 5). Although the uptake rate of tyrosine was similar for both strains, HG001 formed significantly more 4-OH-PLA compared to COL (0.038 mM and 0.013 mM, *p*≤0.01). To our knowledge, this is the first observation of the time-course of the production of 4-OH-PLA by *S. aureus*. Probably 4-OH-PLA is being secreted to avoid an intracellular accumulation of tyrosine, which has also been suggested for lactic acid bacteria [51].

## Conclusions

For the first time, a time-resolved metabolic footprint analysis of two commonly used *S. aureus* strains is presented, monitoring the growth behaviour of this pathogenic bacterium in an infection related culture medium. RPMI 1640 is an established culture medium for a wide range of mammalian cells and provides a pH-controlled environment. Its fixed composition guarantees a well reproducible growth of *S. aureus* COL and HG001, which accompanies reproducible metabolic uptake and secretion patterns for both strains. This confirms RPMI 1640 as a suitable culture medium for laboratory growth experiments of *S. aureus*.

In this study, we are also able to highlight relevant differences between the metabolic footprints of *S. aureus* COL and HG001. The distinct exometabolic profiles are based on the varying accumulation of diverse metabolites in the supernatant, like the intermediates of the central carbon metabolism including the overflow metabolites. Besides, further interesting metabolites accumulated in considerable amounts in the medium, as they are D-isoleucine as a NCDAA and several organic acids as intermediates of the amino acid metabolism. Several metabolites e.g. short-chain fatty acids, including acetate, propionate, butyrate and also yet unknown fermentative products, are postulated to have diverse effects on the metabolism of eukaryotic cell lines [53], [54], [55]. Therefore, the secreted organic acids of *S. aureus* and their yet unknown impact on the bacterial metabolism or on eukaryotic cells, provide interesting starting points for further experiments.

## Supporting Information

Figure S1 **Statistical separation of COL and HG001 based on the exometabolome data during their growth.** (**A**) The loading plot according to the component 1 of the principal component analysis of the extracellular metabolic data of COL and HG001 during their growth in RPMI medium is presented. (**B**) The different glucose uptake rates of COL (blue points) and HG001 (red points) are visualized by plotting the extracellular glucose concentration as a function of growth. (**C**) The different acetate secretion rates of COL (blue points) and HG001 (red points) are visualized by plotting the extracellular acetate concentration as a function of growth. Single values of 4 biological replicates are displayed in B and C, whereby points in light blue and light red display metabolite concentrations during the stationary growth phase when the optical density slightly decreased.
(TIF)

Figure S2 **Extracellular pH value and the acetate concentration.** The pH values of the culture supernatant of both staphylococcal strains during growth in RPMI medium are presented as a blue line (COL) and a red line (HG001). The extracellular amounts of acetate are displayed as blue columns (COL) and red columns (HG001). Data are shown as mean values ± SD of quadruplicate samples.
(TIF)

Table S1 **Absolute metabolite concentrations in mmol/l.** Data are shown as mean values ± SD of quadruplicate samples.
(XLSX)

Table S2 **Relative concentrations of unknown metabolites.** Unknown metabolites are named according to the chemical shift (ppm) of its signal and the signal multiplicity in the NMR spectra. Data are shown as mean values ± SD of quadruplicate samples.
(XLSX)
