# Supplementary figures and images for: Metabolic Footprint Analysis Uncovers Strain Specific Overflow Metabolism and D-Isoleucine Production of Staphylococcus Aureus COL and HG001
Source: PLoS One. 2013 Dec 3;8(12):e81500. doi: 10.1371/journal.pone.0081500 (PMC3849228; doi:10.1371/journal.pone.0081500)

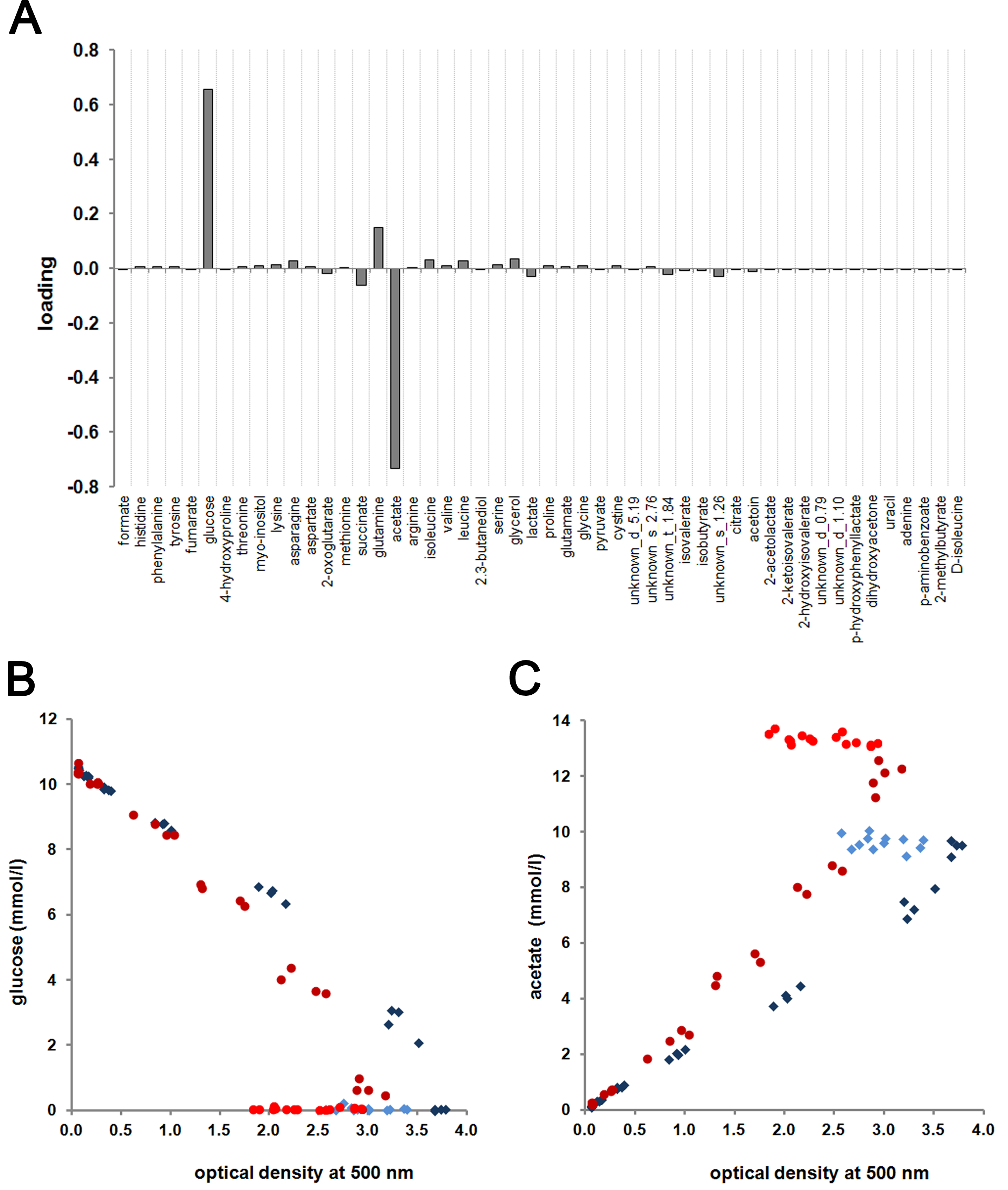

Supplement: Figure S1 — Statistical separation of COL and HG001 based on the exometabolome data during their growth. (A) The loading plot according to the component 1 of the principal component analysis of the extracellular metabolic data of COL and HG001 during their growth in RPMI medium is presented. (B) The different glucose uptake rates of COL (blue points) and HG001 (red points) are visualized by plotting the extracellular glucose concentration as a function of growth. (C) The different acetate secretion rates of COL (blue points) and HG001 (red points) are visualized by plotting the extracellular acetate concentration as a function of growth. Single values of 4 biological replicates are displayed in B and C, whereby points in light blue and light red display metabolite concentrations during the stationary growth phase when the optical density slightly decreased. (TIF) [file pone.0081500.s001.tif]

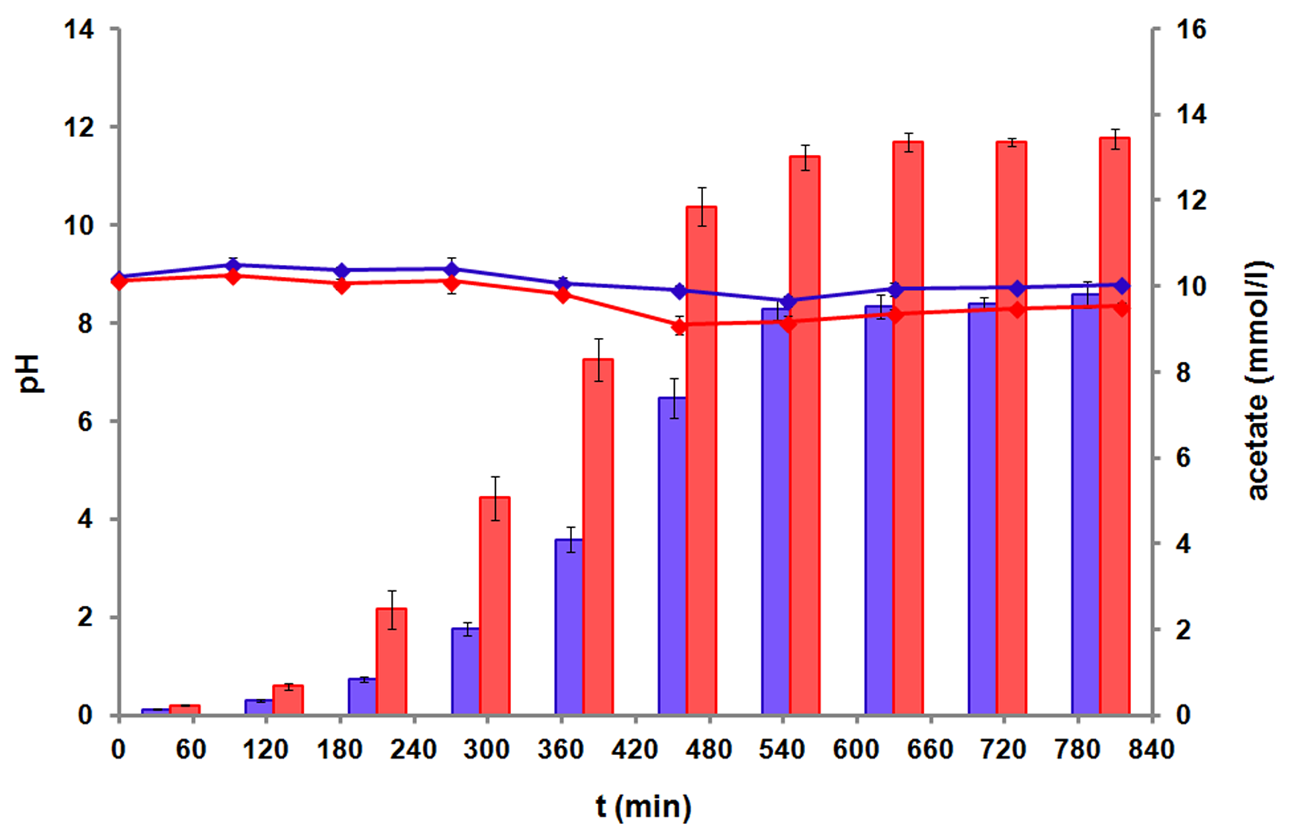

Supplement: Figure S2 — Extracellular pH value and the acetate concentration. The pH values of the culture supernatant of both staphylococcal strains during growth in RPMI medium are presented as a blue line (COL) and a red line (HG001). The extracellular amounts of acetate are displayed as blue columns (COL) and red columns (HG001). Data are shown as mean values ± SD of quadruplicate samples. (TIF) [file pone.0081500.s002.tif]
